# Sooo Sweeet! Presence of Long Vowels in Brand Names Lead to Expectations of Sweetness

**DOI:** 10.3390/bs11020012

**Published:** 2021-01-20

**Authors:** Abhishek Pathak, Gemma Anne Calvert

**Affiliations:** 1 School of Business, University of Dundee, 4 Nethergate, Dundee DD1 4HN, UK; 2 Nanyang Business School, Nanyang Technological University, 50 Nanyang Avenue, Singapore 639798, Singapore; gcalvert@ntu.edu.sg

**Keywords:** product attributes, sound symbolism, vowels, cross-modal correspondence, taste

## Abstract

Throughout the history of languages, poets and writers have used linguistic tools to enhance euphony in their creations. One of the widely used tools to convey melody in any written (or spoken) creative art form is the use of long vowels. This paper examines the linkages between long (vs. short) vowel sounds and taste expectations of sweetness. Across four studies, we demonstrate that people expect products with brand names containing long vowels to taste sweeter than those including short vowel sounds. In studies 1 and 2, we demonstrate this association with the use of self-reported measures, and in studies 3 and 4, we employ indirect measures (implicit taste–shape correspondence and Single Category Implicit Association Test (SC-IAT) paradigm) to show the effect holds at a subconscious level of processing. Previous research in this field has typically linked vowel position (high vs. low or front vs. back) with product or brand attribute expectations. This paper contributes to the growing body of literature in this field by demonstrating the importance of vowel length in sound symbolism, and more precisely, how it pertains to the taste continuum.

## 1. Introduction

*The Lotos blooms below the barren peak*

*The Lotos blows by every winding creek*

*All day the wind breathes low with mellower tone*

*Thro’ every hollow cave and alley lone*

*Round and round the spicy downs the yellow Lotos-dust is blown*

These famous lines from Lord Tennyson’s poem The Lotos-eaters are known for their lullaby-like effect on a listening audience. Across cultures and languages, successful poets often use linguistic tools to enhance euphony and melody in their expression. These include the use of vowel rich language, long vowel sounds, alliteration, rhyme, and soft speech sounds (e.g., /l/, /r/, /w/). This trend has not gone unnoticed by the communications industry and indeed similar practices are observed in the development of creative marketing messages, when advertisers for example, use some of these poetic devices to enhance euphony in their communications (e.g., Grace, Space, Pace for the brand Jaguar) [1].

There is evidence in the linguistic literature to suggest that some sounds are considered euphonic [e.g., vowels (/a/, /e/) and liquids (/l/, /r/)] and others cacophonic [e.g., voiced fricatives (/th/ as in then)]. Euphony is described as an enjoyable sound, the use of which enhances the inherent acoustic pleasantness of words or sentences to achieve a harmonious and melodious effect [2]. The reverse is true of cacophony, which refers to the harsh discordant mixture of sounds. In this paper, we show that when euphonic linguistic tools are used to create brand names, specifically, embedding the long vowels sounds (e.g., the /ea/ sound in the word /peak/ as in the poem, The Lotos-eaters), the expectation that is generated is for a product that tastes sweeter (vs. products with brand names that do not include such melodic sounds). In the current paper, we provide evidence that euphonious phonemes (e.g., long vowels) present in a brand name can lead to expectations of product sweetness. The paper presents a literature review (Section 2, Section 3 and Section 4), method and studies (Section 5 and Section 6), and a general discussion, managerial implications, and limitations (Section 7).

## 2. Literature Review

A large body of research has now established a link between the linguistic features contained within a word (or a brand name) and product attributes (e.g., speed, taste, luxury, aggression, harshness, angularity, creaminess, femininity, likeability, size [3,4,5,6,7,8,9,10,11,12,13,14,15,16,17,18]). Of specific relevance to the current paper, both vowels [18,19,20] and consonants [8,13,14] have been shown to influence taste expectations and perception. Similarly, Spence and colleagues (e.g., [21,22,23,24]) have linked music or musical notes to the taste continuum. Despite the considerable body of research on this topic, most findings to date relate to the connection between taste and the vowel distinction based solely on their articulatory position (e.g., how front vs. back vowels or low vs. high vowels modulate taste perception). In contrast, the impact of vowel length (i.e., short vs. long vowel sounds) on taste expectations remains largely unexplored, including in the area of consumer research.

In the English language, there are five vowels, each with a corresponding long or short vowel sound (e.g., bug vs. bugle). Long vowels are almost always the first sounds produced by babies and are believed to be melodious, and hence widely used by poets and writers alike as euphonic tools. It seems plausible, therefore, that the inclusion of long vowel sounds in brands’ names would enhance their pleasantness and modulate expectations of their corresponding products in predictable ways. Such a phenomenon, if proved true, would be of considerable interest to marketers and brand managers alike.

## 3. Speech and Phonemic Sounds

Speech is a complex acoustic stimulus [25]. Broadly, speech sounds can be categorized into vowels and consonants, where both are believed to have different roles in the communication process [26]. Most languages contain more consonants (vs. vowels), which are typically the main provider of the lexical root in a word because they sound so distinct (compared to vowels) [27]. Vowels, on the other hand, highlight the finer differences within the lexical information conveyed by the consonants [27]. For example, in Arabic, the lexical root for the concept ‘to write’ is provided by the consonants /k/, /t/, and /b/ and different variations related to the concept of writing are provided by just altering the vowels (e.g., /kitab/ = book, /kataba/ = he wrote, /kutiba/ = it was written etc.). The importance of consonants in word recognition is evident by the observation that if consonants are retained and vowels deleted, speakers can still guess the word correctly. The same is not true in reverse (e.g., in the word ‘direction’, if consonants are deleted then the original word can still be guessed from the remaining root /drctn/, but if only vowels are retained, i.e., /ieio/ it is almost impossible to guess the original word) [27]. While consonants carry lexical information, vowels provide the acoustic cues, melody, and prosody within speech [27,28,29,30]. Indeed, vowels are said to be the most relevant amongst voiced speech sounds [31]. In the English language, 78% phonemes are voiced [32] and are particularly vital in speech communication [33].

Research has shown that people communicate extensively using prosody alone (e.g., by using stresses, duration of sounds, pitches etc.) and vowels are especially good at conveying different speech cues (e.g., emotions, sarcasm, irony, contrast) by prosodic manipulations. To illustrate, the phrase “He plays piano” (factual) vs. “He plays piano?” (inquisitive) can be communicated as distinctive, simply by changing the prosody [34]. Similarly, the phrase “The food was great”, could be conveyed as sarcastic or genuine, depending on the prosodic information conveyed by the vowels in speech. Further, nouns can be differentiated from identically spelled verbs [e.g., the word ‘present’ can refer to a gift (noun) or to deliver (verb)] simply by adapting the prosody [25].

People also use prosodic information to convey traits, which they are referring to in sentences. For example, when people talk about upwards (vs. downwards) motion, they tend to raise (or lower) their pitch accordingly [35]. Similarly, when people describe speed (e.g., how fast a car can go) the pace of their speech increases. This contrasts with the slowing of speech sounds when describing slow moving objects, e.g., “a sloooow truck” [35,36]. Vowels also convey emotions. For example, by changing a vowel in the hypothetical word ‘jVj’ (where v is the vowel), the emotion perceptibly signified by the word has been shown to change (i.e., different emotions are felt for the words /jaj/, /joj/, and /juj/) [37]. The information conveyed by prosody can also be looked as a ‘spoken gesture’ [38] and since listeners can extract a wealth of information from a ‘speaker’s gestures’, so can they from the spoken gestures or prosody.

In fact, the use of vowels to convey speech cues is not only observed in adult human speech but also in primate and infant communication. In primate communication (which consists mostly of ‘vowel-like’ sounds), the information about the affective state (e.g., positive vs. negative, danger vs. pleasant) is effectively conveyed by prosody [39]. The same is true of human infants who use vowel-laden coos and aahs (prosodic manipulations) to communicate with their mothers [40,41]. Even people with speech disorders are known to use prosody to communicate [42].

## 4. Euphony, Long Vowels, and Usage

Sound symbolism suggests that, apart from the lexical meanings of words, sounds contained within a word itself convey various meanings to a listener. Some sounds are acoustically harsh. These are predominantly consonant sounds (e.g., those embedded in the onomatopoeic words “crash” and “crunch”). Others are more pleasing to the ear. These are primarily vowel sounds, such as those contained in the words “soak” and “wallow” [43]. Among speech sounds, vowels and sonorants (e.g., /l/, /r/) are known to introduce a melodic tone into a language, and the more numerous their inclusion in a word or sentence, the more melodious is the perceived sound [44]. The association of long vowels with euphony has not only been shown in the English language, but even in other languages (e.g., in Arabic where vowels are referred to as the “sounds of softness” [45]).

The use of melody (and euphony) is also used in motherese (also known as the infant directed speech), a peculiar way in which mothers communicate with their babies [46]. Motherese has a higher pitch, slower speed, extra stresses, and exaggerated intonations [47]. In addition, motherese is especially rich in vowels (often called as vowel drenched [46]), with a higher usage of long vowels and prolonged short vowels [48], all of which add to the sweet quality of motherese. Similarly, research suggests that lullabies have a soothing effect on babies due to the presence of long vowels, phonemic repetitions, soft sounds, and rhyming (i.e., tools similar to the ones used by poets) [49,50]. Both motherese and lullabies bear similar phonetic structures across many cultures and languages (e.g., long vowels, slow speech, exaggerated sound patterns), which underlines their universal appeal.

The use of long vowels to induce melody is not exclusive to motherese. Even in adult speech communication, 75% of adults admitted to having used baby talk in their relationships [51]. When using baby talk, couples often use phonemic patterns similar to those used in motherese, often containing made-up words and endearments mostly with long vowels (e.g., sweetie, beddiebye) [51]. Research suggests that many adults find such talk rather cute [51] and baby talk has even been shown to strengthen couple relationships [51]. Researchers feel that adults use baby talk, (1) when they want to appear warm and personal to the other person, (2) to signify affection and attachment, (3), to show intimacy and affection, and (4), to be more expressive [51,52] and like motherese (and lullabies), baby talk between adult couples has been observed across numerous cultures.

Although we are not explicitly aware of it, most of us can extract meaningful information from a name alone. For example, people can often differentiate an unknown female name from a male one based purely on their phonemic patterns. This is because across cultures, female names are generally longer, contain more vowels and long vowels and have more initial or name-ending vowels than male names [53,54,55].

Similarly, research in the academic marketing literature has shown that when consumers encounter unknown brand names, they often rely on the phonetic signals conveyed by a brand name to gain an impression of the brand or the associated product. More precisely, consumers anticipate a level of congruency between the brand name and its related product line or brand attributes. For example, if a brand name sounds ‘rugged’, consumers expect that the product or brand will itself be tough. In other words, that brand or product might be suitable for a rugged or adventurous activity [56]. Similarly, if the brand name sounds harsh, consumers often associate that brand with harsher versions of the same product (e.g., hard vs. soft toilet cleaner and strong vs. mild beer) [8,10]. With respect to the current paper, this association has also been shown to hold true on the taste continuum. For example, previous research has shown that a chocolate brand name containing harsh speech sounds leads to higher expectations of bitterness compared to those containing soft sounds [8]. If harsh speech sounds are perceived as rugged and bitter (e.g., [8,56]), it seems plausible that melodious speech sounds would be associated with perception of sweetness.

We propose to use the transitivity hypothesis as the basis of this prediction. According to the transitivity hypothesis, consumers will link two stimuli (e.g., A and B) together if those stimuli are linked with another common stimuli (e.g., C) (i.e., if A is linked to C and B is linked to C, then A will become linked to C by associative learning). Deroy, Crisinel, and Spence [57] have previously used the transitivity hypothesis to explain cross modal linkages between various sensory pathways. For example, if round and curved objects are considered pleasant [58] and sweet tastes are perceived pleasant, then sweet tastes will become linked with roundedness [59]. Similar crossmodal linkages have been shown with olfactory, auditory, and taste combinations. Using this as the basis, we propose that if long vowels are perceived as euphonic and pleasant, and sweet tastes are pleasant, then words with long vowels should be perceived as sweeter than words containing short vowels. Specifically, we predict that when long vowel sounds are embedded in fictitious brand names, they lead to greater expectations of sweetness than brand names with short vowel sounds. At present, there is scant research linking vowel length (short vs. long) with product attributes and expectations. The current research contributes to the extant literature on sound symbolism, taste continuum, and product attributes.

## 5. Methods and Overview of Studies

Ten hypothetical brand name (HBN) pairs (see Appendix A) were created in a bi-syllabic (CV-CVC; consonant-vowel-consonant) format using thirteen consonants (/s/, /k/, /t/, /z/, /m/, /l/, /g/, /n/, /v/, /r/,/ /h/, /f/, /p/) and five vowels (/a/, /e/, /i/, /o/, /u/). Each HBN pair differed only in the type of the vowel used, i.e., short vs. long [e.g., HBN /gelin/ (short vowel: IPA notation /ɡelɪn/) vs. (long vowel: IPA notation /ɡiːlaɪn/)]. The HBNs were converted to auditory format (in an American accent) using Google’s text to speech conversion. The experiments were created on the Inquisit 5 platform (Millisecond.com) and native English speakers were recruited from the USA through the Amazon Mechanical Turk [60] to participate in the studies. Participants were allowed to take part in only one study related to the current paper.

Our aim was to test the association of long vowels with sweetness (compared to short vowels). In Study 1, participants rated the HBNs of chocolates on a Likert scale (less sweet to very sweet) and a *t*-test was used to compare the mean differences in Likert ratings. In Study 2, respondents were instructed to generate HBNs for sweet and very sweet chocolates with a given set of consonants, and the number of vowels (short vs. long) used by participants in creating the HBNs were analyzed. A Wilcoxon signed-rank test was used to compare the proportions of long vowels used for creating BNs for very (vs. less) sweet chocolates. While studies 1 and 2 used self-reported measures, studies 3 and 4 aimed to test the association of HBNs with sweetness indirectly. In Study 3, we tested this association using the shape–taste correspondence (e.g., sweetness with floral vs. round/square shapes). A Wilcoxon signed-rank test rank test was used to compare the proportions of HBNs (long vs. short vowels) with shapes (floral vs. circular). Since phonetic symbolism is known to be automatic and non-conscious, Study 4 explored the association using an implicit association test. D scores and *t*-tests were used to compare the response latencies and the implicit association of phonemic sounds with respective taste attributes. Across all studies, respondents were required to guess the purpose of the experiment. If they guessed correctly, their data was excluded and reported in the results.

## 6. Experimental Studies

### 6.1. Study 1: Analyzing the Likert Ratings of the HBNs

#### 6.1.1. Participants

Sixty-one participants between the ages of 22 to 65 years completed the study (M _age_ = 34.75 years, SD = 10.16, Males = 37, Females = 24). All participants were native English speakers (three participants also knew Spanish and one knew French).

#### 6.1.2. Procedure and Design

Participants were informed that a well-known brand was launching two new chocolates (one less sweet than the other) in an international, non-English speaking market and seeking suitable brand names for them. Participants were told that the company desired a BN where consumers could judge by the name alone whether the chocolate was a very sweet tasting one or one that was considerably less so. They then randomly heard all of the twenty HBNs (one at a time) and rated them on an eleven-point Likert scale (1 = ‘less sweet’ and 11 = ‘very sweet’; HBNs were continuously played on a loop until a response was received).

#### 6.1.3. Results and Discussion

Results show that participants rated HBNs with long (vs. short) vowels as more appropriate for a very (vs. less) sweet chocolate, (M _Long vowels_ = 6.18, SD = 1.08, M _Short vowels_ = 5.66, SD = 1.26, t (60) = 2.82, *p* = 0.006, d = 0.36). The results of Study 1 support our hypothesis and provide evidence (with a medium effect size) for a link between brand names with long vowels and expected taste attributes (i.e., higher sweetness). Specifically, it demonstrates a link between the long vowels and sweetness (when compared to short vowels). We are not claiming that short vowels are not expected as sweet but are showing that long vowels are perceived to be sweeter than their shorter counterparts and can be used to enhance the expected sweetness of a food product.

### 6.2. Study 2: Analyzing the Proportions of Vowels Used to Create the HBNs

While Study 1 provides support for the hypothesis that the inclusion of long vowels in HBNs enhances the expectation of a sweeter chocolate than short vowels, it could be argued that the requirement for respondents to provide explicit ratings may not be representative of natural language usage. To further understand the boundary conditions under which this long vowel-high sweetness expectation persists, we conducted a second study, which used a more naturalistic paradigm. In Study 2, we asked participants to create their own HBNs in a free choice task and examined the vowels they used to create brand names signifying less vs. more sweet products. If this sound symbolic taste phenomenon is not bound by paradigmatic context, then the results should demonstrate the higher use of long (vs. short) vowels for brand names representing sweeter products.

#### 6.2.1. Participants

Sixty-two participants between the ages of 24 to 71 years completed the study. Two participants guessed the hypothesis to some extent and their data were excluded from the analysis (M _age_ = 40.65 years, SD = 10.61, Males = 30, Females = 30). All participants were native English speakers (four participants also knew Spanish, two knew German, and one knew Western Armenian).

#### 6.2.2. Procedure and Design

Participants were provided with the same storyline as in Study 1 but were instead asked to create their own brand names corresponding to chocolates that were sweet or less so. They were then shown a brand name pair where the consonants were in the same CV-CV-C format as in the supplied HBNs in Study 1 (e.g., Z_M_L), but this time participants had to choose vowels (from short vowels- /a/, /e/, /i/, /o/, /u/ and long vowels- /a:/, /e:/, /i:/, /o:/, /u:/) to create three brand name pairs (one BN each for the less (vs. very) sweet chocolate). Before the experiment began, participants were familiarized with the experimental procedure and the vowel symbols (long and short) by using real English words with long vowels (e.g., mute, mite, meat) in a few practice trials.

#### 6.2.3. Results

**Figure 1:**
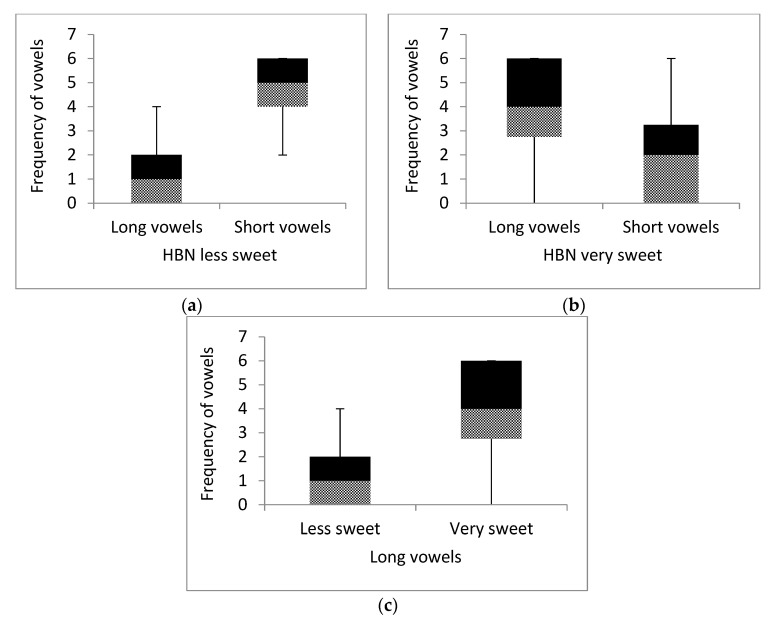
Frequency of long vs. short vowels in Hypothetical Brand Names created by participants in Study 2. (Boundary of the black and patterned box demarcates the median; black box shows 3rd quartile-median and patterned box shows median-1st quartile).

A Wilcoxon signed-rank test compared the frequencies of vowels (short vs. long) used by participants to create the HBNs for less (vs. very) sweet chocolates. Results showed that participants chose a significantly higher number of short (vs. long) vowels for creating BNs for less sweet chocolates, Z = 6.48, *p* < 0.001, r = 0.59 [Figure 1a] and a significantly higher number of long (vs. short) vowels for creating BNs for very sweet chocolates, Z = 3.83, *p* < 0.001, r = 0.35 [Figure 1b]. Specifically, we are more interested in comparing the long vowels used across sweeter (vs. less sweet) categories. Participants used significantly higher number of long vowels (with a large effect size) for creating BNs for very (vs. less) sweet chocolates, Z = 5.86, *p* < 0.001, r = 0.53 [Figure 1c].

### 6.3. Study 3: Analyzing the Indirect Association of Shapes with Taste Attributes

In Study 3, we aimed to demonstrate the linkage between long vowels and expectations of sweetness using a well-established shape–taste correspondence paradigm (i.e., by using indirect means). A large body of research has demonstrated cross modal linkages between shapes (both abstract forms such as “round” vs. “spiky”, as well as concrete shapes used in food packaging) and taste (e.g., sweetness with roundedness and spikiness with sourness or bitterness) (see [17] and [61] for a review). Chocolates too come in all shapes and sizes. In the first part of this study (3a), we explored and selected chocolate shapes, which would be considered as ‘sweetest’ by respondents. In the second part of the study (3b), we used these shapes (rated by the first cohort of participants as most likely signifying sweet chocolates vs. less so) to test whether the HBNs with long (vs. short) vowels would be matched with these shapes (or not). If participants considered HBNs with long vowels as ‘sweeter’, then the association could be discernible in the shape–taste correspondence.

#### 6.3.1. Study 3a

##### Participants

Fifty-one participants between the ages of 25 to 69 years (M _age_ = 37.78 years, SD = 10.41, Males = 17, Females = 34) completed the study.

##### Procedure and Results

Participants were told that different shaped chocolates would be presented to them on a computer screen and they would be asked to decide which one would be the sweetest. They were then shown four chocolate shapes (floral, circle, square, and triangle). All four shapes were shown at the same time and the order of presentation was randomized between participants. Participants clicked on the shape, which they felt was the sweetest among the chocolate shapes.

Results revealed that most participants felt that a floral shaped chocolate would be sweetest (60.78%), followed by circular shape (28.41%), square shape (9.80%) and none of the participants chose the triangular shaped chocolate. As we wanted to choose two shapes of chocolate for Study 3b, we selected the floral and circle shapes (as these were chosen as sweetest); also, differences between these were significant, χ^2^ (1, *n* = 46) = 5.56, *p* = 0.018, w = 0.35 (no gender differences were found).

#### 6.3.2. Study 3b

##### Participants

Sixty-one participants between the ages of 19 to 68 years completed the study (M _age_ = 39.18 years, SD = 10.52, Males = 27, Females = 34). All participants were native English speakers (four participants knew Mandarin, Bulgarian, French, and Dutch).

##### Procedure and Design

Participants were told that they would hear an unknown word in a foreign language, which would refer to one of the images shown on the screen (a floral and a circular shape) and were asked to click on the image which they thought the foreign word referred to. Participants then heard all the 20 HBNs in a randomized fashion. Before the study began, participants were familiarized with the experiment in a few practice trials with known words of English. We predicted that participants would associate HBNs containing long vowel sounds more with the floral shape than with the circular shape.

##### Results

A Wilcoxon signed-rank test was used to compare the proportion of HBNs (long vs. short vowels) with shapes (floral vs. circular). Results show that participants associated HBNs _Long vowel_ with the floral shape significantly more (with a medium effect size) than with the circular shape, (M _Floral_ = 5.77, SD = 1.63, M _Circle_ = 4.23, SD = 1.63, Z = 3.34, *p* = 0.001, r = 0.30). Participants associated floral shape more with HBNs _Long vowel_ than with the HBNs _Short vowel_, Z = 3.23, *p* = 0.001, r = 0.29. The association of HBN _Short vowel_ with the floral shape was found to be weaker than with the circular shape (although this was not significantly different) (M _Floral_ = 4.64, SD = 1.52, M _Circle_ = 5.37, SD = 1.52, Z = 1.80, *p* = 0.07).

The results of Study 3 suggest a stronger association of HBNs _Long vowels_ with floral shapes (Study 3b), which are also perceived to be sweeter (Study 3a). Results further support our claim of the linkages between long vowel and sweetness by using the indirect shape–taste correspondence.

### 6.4. Study 4: Analyzing the Implicit Association of Vowels with Taste Attributes Using IAT

Phonetic symbolism has been shown to occur automatically at a non-conscious level of perceptual processing. In Study 4, we extend our findings to show that the association between long vowel sounds and the expectation of sweetness operates at an implicit level by using a well-established paradigm, the Single Category Implicit Association Test (SC-IAT [62]). Here, we explored the implicit association between speech sounds (e.g., long vowels) with certain taste attributes (e.g., sweetness). The Implicit Association Test (IAT) [63] is widely used in cognitive and behavioral research to measure implicit associations between certain categories (e.g., insects vs. flowers) and specific traits or attributes (e.g., threatening vs. pleasing) by analyzing reaction times in a speeded classification or sorting task. The SC-IAT is a modified version of the IAT and permits measurement of the strength of association between a single category and two attributes or objects. Research in this field has used similar other modifications of the popular IAT (e.g., Single Target IAT or ST-IAT [64]) to address slightly different empirical questions. The IAT has also been used in the past with auditory (rather than visual) stimuli to test cross-modal associations in the sensory domain (e.g., [65,66]).

#### 6.4.1. Participants

Thirty participants between the ages of 22 to 73 years took part in the study (M _age_ = 37.83 years, SD = 11.43, Males = 13, Females = 17). All participants were native English speakers (four participants also knew Spanish and one knew Russian).

#### 6.4.2. Procedure

A single category IAT [62] was used and the script for the experiment was downloaded and modified from the script library of Millisecond.com. Participants were informed that a well-known brand owned two types of products: sweet (e.g., jam) vs. non-sweet (e.g., bread) and was looking for new brand names for its sweet products. The company desired that the sound of the brand names chosen should themselves provide a clue about the associated product’s sweetness. Participants were then asked to complete the task where they had to categorize brand names (auditory HBNs _Long vowels_) and products (sweet vs. non-sweet). All ten HBNs _Long vowels_ were used as the auditory stimuli and the products related to two attributes were either sweet vs. non-sweet [sweet products (jelly, jam, honey, caramel, chocolate, sugar, custard, candy, cake, cookie) and non-sweet (bread, sausage, hotdog, burger, salad, eggs, crisps, pretzel, chips, pizza)].

In the categorization task, participants had to press the “E” key on the keyboard if HBNs or sweet products were presented and the “I” key press when non-sweet products were presented. The pairings (i.e., HBNs with sweet products) were reversed in the next block (i.e., in the next block, HBNs with non-sweet products were presented as a pair). As per the SC-IAT script, there were two main test blocks of 72 trials each, one compatible with the hypothesis (i.e., HBNs _Long vowels_ with sweet products) and the other incompatible (i.e., HBNs _Long vowels_ with non-sweet products). Before each test block, a practice block of 24 trials (as in the original SC-IAT) was presented, which aimed to familiarize the participants with the test procedure (practice block responses are not counted towards the calculation of the final results or the D score). Throughout the experiment, the pairing for the keys was displayed on top of the screen (e.g., in the compatible block, ‘Sweet OR Brand names’ was displayed above the E key and ‘Non-sweet’ was displayed above the I key and vice versa); the order of the key mapping was counterbalanced between participants.

#### 6.4.3. Results

The results were analyzed in two ways, (1) following the approach of scoring suggested by Greenwald et al. [67], and (2), comparing the response latencies and error rates in compatible vs. incompatible blocks (as in [66]). D scores are computed as the mean difference between compatible and incompatible blocks, divided by the pooled standard deviation. In Study 4, the D-scores were automatically calculated by the script using the improved algorithm, and the error trials were handled by requiring the respondents to correct their responses (both procedures as described by Greenwald et al. [67]). Two participants had more than 70% response latencies at less than 300 ms and their data was excluded.

In SC-IAT, the strength of association between category and attribute is measured by the standardized mean difference score of compatible (or hypothesis consistent) vs. incompatible (hypothesis inconsistent) blocks [67]. A higher D score suggests stronger association between the hypothesis-consistent pairings (and vice versa for the negative D scores). A one-sample *t*-test on the D values showed significant differences from zero (M = 0.18, SD = 0.38, t (27) = 2.48, *p* = 0.02, d = 0.47), which shows stronger association (with a medium effect size) of HBN _Long vowels_ with sweet products than with non-sweet products.

Paired-sample *t*-tests of response latencies revealed significant differences (though borderline) between compatible vs. incompatible blocks (M _Compatible_ = 769.39 ms, SD = 166.61, M _Incompatible_ = 814.32 ms, SD = 162.53, t (27) = 2.06, *p* = 0.049, d = 0.39) which suggests that participants were faster (with a medium effect size) in associating sweet products with the HBN _Long vowels_ (than with the non-sweet products). Error rates were higher in the incompatible block vs. the compatible block though not significantly different (Error rate data are non-normal in distribution; Paired *t*-tests and the means of error rates are reported here as these are robust and are more relevant here than the medians (similar to [48]). The analysis with non-parametric tests also shows similar results.) (M _Compatible_ = 5.4%, M _Incompatible_ = 4.3%, t (27) = 1.65, *p* = 0.11). Overall, the results of Study 4 support the association of HBN long vowel with sweetness using an indirect and implicit measure.

## 7. General Discussion

Vowels are the main tools to convey prosodic information in speech and music [68] and the use of long vowels to enhance melody and sweetness is common amongst artists (e.g., opera singers are known to shorten consonants and prolong the vowels in order to enhance the melody in their singing) [69,70,71]. In fact, some scientists go so far as to remark that “vowels sing but consonants speak” [28]. Since long vowels can enhance melody in singing, they can convey sweetness in a word or name, which we demonstrate in the current paper. In the context of the current paper and cross-modal correspondences within the taste continuum, research has shown that the lexical root that signifies the meaning of a taste in speech can be delivered by any arbitrary consonant sound (e.g., /s/, /w/ /t/ for /sweet/ in English; /d/, /x/ for /doux/ (meaning sweet) in French). But how do we convey qualitative or finer differences within the different taste dimensions (e.g., sweet vs. very sweet)? It is unlikely that these distinctions can be sensorially conveyed through the use of different consonants to describe the same taste (e.g., different words for describing less vs. medium vs. very sweet). As highlighted in the section above, most languages rely on prosodic manipulations to convey more fine-grained sensory distinctions. Against this backdrop, we hypothesized that since long vowels are more melodious and euphonic, they may be used by speakers to convey sweetness (i.e., sweeter than words containing short vowels). According to the transitivity hypothesis, consumers often link two stimuli together, if they are linked by common stimuli, and across four studies, we demonstrate that people do indeed associate brand names containing long vowels with sweetness significantly more so than brand names with short vowels. In Studies 1 and 2, we used explicit, self-reported measures to arrive at these findings and in Studies 3 and 4, we demonstrate that the phenomenon persists even with the use of indirect measures, as shown from the results of the taste–shape correspondence paradigm and the SC-IAT.

### 7.1. Managerial Implications

The advancement of globalization has witnessed ever more big-name brands expanding into linguistically diverse markets. In the past, household brands have been reluctant to change their names, but not anymore. Increasingly, brands are taking advantage of the local linguistic strengths while renaming for international markets [72]. Well known examples include Coca-Cola changing its name to Ke-kou-ke-le in China [73] and Toyota changing its tagline to ‘Akeed’ (meaning “sure” or “yes of course” and used often by younger people) to engage young Middle Eastern customers [74].

Certain linguistic features of one spoken language may be considered harsh sounding or difficult to pronounce in other cultures or languages (e.g., /l/and /r/ in Japanese) and brand managers need to consider changing such features in their brand names to appeal more to local consumers. This is important since sound symbolic congruency in names and product or brand features has been shown to be a significant factor in product evaluation, purchase decision, and even post purchase evaluations [7,9,11,75,76]. To illustrate, the name Haagen Dazs has five consonants (one silent), out of which three (/g/, /d/, and /z/) are referred to as voiced obstruents which are considered to be particularly harsh sounding when pronounced [8,10,13,14]. The presence of multiple voiced obstruents do, however, connote luxuriousness as they are rarely encountered amongst household or necessity product brand names and are more often embedded into brand names in the luxury market [9]. While Haagen Dazs is clearly a highly successful brand in the premium ice cream category, similar sound symbolic incongruence might be best avoided in new brand names, especially for sweet tasting products (such as chocolates or ice creams) in the basic foods category.

As with any other word, the linguistic information contained in brand names is provided by the consonants, whereas the qualitative meaning is further conveyed by vowels and the prosodic manipulations [77]. The consonants in a word can be quite arbitrary and different. For example, a fruit may be called an Apple (English), a Pomme (Canadian) or a Mello (Greek), all of which are acoustically distinct, and yet all of them contain soft and pleasant sounds (e.g., /m/, /l/). Although these words may sound different in different languages, sound-symbolically these names still convey softness and sweetness [78], which is similar to the reason as to why brands desire sound symbolic congruency in their names.

Names can also convey emotional information [78,79] (e.g., boys nicknames are typically humorous, whereas girls’ ones are considered warmer and more affectionate [80]). Similarly, nicknames related to mothers have soft sounds across cultures e.g., /m/, /n/ as in /mama/, /nana/ or just vowel sounds in certain languages (e.g., /Aayee/ in the Marathi language). Brand names, slogans, jingles, taglines, and other advertising materials use linguistic tools to connect with consumers in a more engaging and memorable fashion. Research suggests that both advertising and poetry exploit similar tools [81] and a more condensed language for greater impact on their listeners [82]. Indeed, some scholars even refer to advertising as the poetry of commerce. A few famous examples include, “Beanz meanz Heinz”; “Less Flower. More power” and “A Mars a day helps you work, rest and play”. All of these slogans demonstrate the effective use of poetic elements employed by brands to ensure their messages worm their way into consumers’ long-term memory store. In today’s social media rich environment, the use of poetic devices is rife and can be observed in songs, names, text messages, slogans, jingles, brand taglines, and creative campaigns [83,84]. Indeed, branding itself has been referred to as poetry in motion [83] and the more poetic a brand name, the more memorable it becomes [83,85]. Euphony is poetry (e.g., especially that produced by long vowel sounds [86]) and confers an inherent sweetness to names. In this paper, we have demonstrated how brand managers can use these in their brand names to signify sweetness in their products.

Of related interest, while we are recommending the use of long vowels to convey sweet product attributes, another interesting linguistic phenomenon emerging in the marketing field is disem-vowelment, where new brands are omitting vowels altogether from their names to appear cool or stand out (e.g., Flickr, Tumblr, Scribd and even some taglines such as ‘GVE & B KND’). This trend began as many domain names were already taken and brands realized that even by dropping the vowels in the brand name, it would still be recognizable. For example, the pronunciation of the brand Tumblr is not affected phonemically. Whether this trend is set to continue remains to be seen, but what is apparent is that the increasing use of linguistic variations and tools make brand names distinctive, memorable, and catchy, all of which are desired by any brand [87].

### 7.2. Limitations and Future Research

One potential limitation of the current paper is the use of bi-syllabic names as the test stimuli. Of course, brand names range from monosyllabic (e.g., Ford), to quadrisyllabic (e.g., Lamborghini) or can be even longer. Whether our findings generalize to brand names of all lengths is a topic for future research although we have no reason to suspect that the effect will not persist across brands of differing lengths. Similarly, in this series of studies, we used a fixed number of long vowels (i.e., two) across our stimuli. We have yet to research if there is an incremental effect of long vowels in enhancing the expected sweetness of a brand name (i.e., one or three vowels in the same BN). While we have used two different vowels in each stimulus, across languages there are many words (and names) with same vowels (e.g., /banana/). So again, whether our finding will be equally applicable to such names needs further testing. Another limitation is that we have used different set of consonants in the HBNs; it is likely that consonants themselves connote different tastes (e.g., /z & v/ vs. /g & t/) and may be considered differently ‘sweet’ in an HBN. Similarly, the use of different sets of consonants in the HBNs may have led to some of the HBNs having an unduly larger effect on the results (compared to HBNs created from different set of consonants). With the current research design, delineating the finer effects of consonants is not possible; future researchers may control for consonants while creating the HBNs. Lastly, in everyday parlance, long vowels are sometimes used to convey size or extent (e.g., big vs. small). For example, a child is more likely to say ‘a biiiig balloon’ to refer to a giant-sized balloon. This suggests one can lengthen long vowel sounds further to convey extra sweetness [i.e., sweet vs. sweeeeet (for very sweet)]. That said, the reverse seems unlikely for the trait of bitterness [i.e., bitter vs. biiiiitter (very bitter)] as the word employs short vowels instead. Nonetheless it an interesting phenomenon which is intriguing and for further exploration.

## Data Availability

Data is available on request to the corresponding author.

## Appendix A

**Table A1:** Hypothetical Brand Name.

HBN	IPA Notation (Short Vowel)	IPA Notation (Long Vowel)
Sokit	sɒkɪt	səʊkaɪt
Zemal	zæmel	zeɪmiːl
Gelin	ɡelɪn	ɡiːlaɪn
Varum	værʌm	veɪruːm
Kinez	kɪnez	kaɪniːz
Hulim	hʌlɪm	huːlaɪm
Timen	tɪmen	taɪmiːn
Latiz	lætɪz	leɪtiːz
Meliz	melɪz	Miːlaɪz
Fepal	fepʌl	fiːpuːl
